# New insights from MRI-guided laser interstitial thermal therapy for refractory epilepsy: a state-of-the-art overview

**DOI:** 10.3389/fsurg.2026.1642054

**Published:** 2026-05-29

**Authors:** Carlos Quispe-Vicuña, Fernando Terry, Miguel Cabanillas-Lazo, Alejandro Enríquez-Marulanda, Niels Pacheco-Barrios, Forough Yazdanian, J. Pierre Zila-Velasque, David R. Soriano-Moreno, Christian Moran-Mariños, Evan Luther, Lekhaj Daggubati, Martin Merenzon, Jaime Lopez-Calle, Jorge G. Burneo, Ricardo J. Komotar, Ziev B. Moses, John D. Rolston, Carlos Alva-Diaz

**Affiliations:** 1 Grupo de Investigación Neurociencias, Metabolismo, Efectividad Clínica y Sanitaria (NEMECS), Universidad Científica del Sur, Lima, Perú; 2 Department of Neurosurgery, Beth Israel Deaconess Medical Center, Harvard Medical School, Boston, MA, United States; 3 Facultad de Medicina Humana, Universidad de San Martín de Porres, Lima, Perú; 4 Department of Neurosurgery, Brigham and Women Hospital, Harvard Medical School, Boston, MA, United States; 5 Facultad de Medicina Humana, Universidad Nacional Daniel Alcides Carrión, Pasco, Perú; 6 Escuela de Medicina, Universidad Peruana Unión, Lima, Perú; 7 Unidad de Investigación en Bibliometría, Vicerrectorado de Investigación, Universidad San Ignacio de Loyola, Lima, Perú; 8 Department of Neurological Surgery, Leonard M. Miller School of Medicine, University of Miami, Miami, FL, United States; 9 Department of Neurological Surgery, George Washington University, Washington, DC, United States; 10 Department of Neurosurgery, Johns Hopkins University School of Medicine, Baltimore, MD, United States; 11 Department of Neurosurgery, Clínica Internacional, Lima, Perú; 12 Epilepsy Program, Department of Clinical Neurological Sciences, Western University, London, ON, Canada

**Keywords:** evidence synthesis, evidence-based medicine, laser ablation, minimally invasive neurosurgery, refractory epilepsy, thermalcoagulation

## Abstract

**Introduction:**

Recently, MRI-guided laser interstitial thermal therapy (MRgLITT) has emerged as a therapeutic option for drug-resistant epilepsy (DRE). However, the absence of standardized clinical guidelines remains a significant issue.

**Objective:**

To conduct a systematic review of systematic reviews to provide a descriptive assessment of the quality of evidence regarding the application of MRgLITT for DRE.

**Methods:**

We conducted a comprehensive search in PubMed, Embase, Scopus, Web of Science, Cochrane Library, and Google Scholar up to May 30th, 2024. We evaluated the risk of bias in each study using the AMSTAR-2 tool. The primary outcomes assessed were seizure freedom and perioperative or postoperative complications rate, while the secondary outcome was reoperation rate. The certainty of the evidence was appraised using the Grading of Recommendations, Assessment, Development, and Evaluations (GRADE) approach. Finally, pertinent findings were illustrated in a bubble plot.

**Results:**

A total of 16 systematic reviews met eligibility criteria, just including observational studies (cohorts and case series). Fourteen SRs scored “Critically low” with a median 8 [IQR: 5.25–8.25]. Seizure freedom rate ranged from 18.87% to 75.86%, and the most frequent cause of DRE was mesial temporal lobe epilepsy. The most common complications were visual field deficit (2.17%–7.5%) and intracranial hemorrhage (0.96%–8.6%). All the outcomes reported a “very low” certainty of evidence, as randomized studies were absent in the reviews.

**Conclusion:**

This overview provides comprehensive evidence on the application of MRgLITT for DRE). The evidence indicates a seizure freedom rate ranging from 18.87% to 75.86% with certainty levels raning from critically low to low. While these findings are promising, further primary and secondary studies are essential to establish definitive conclusions and assess the potential for future clinical applications or recommendations.

**Protocol Registration Number:**

CRD42022339024.

## 1 Introduction

Epilepsy is a chronic non-communicable disease affecting the central nervous system (CNS), characterized by refractoriness in approximately 30%–40% of patients (1, 2). Nonetheless, following the definition established by the International League Against Epilepsy (ILAE) Task Force (3), the pooled incidence and prevalence proportions of drug-resistant epilepsy (DRE) are 0.15 (95% CI: 0.11–0.19) and 0.30 (95% CI: 0.19–0.42), respectively (4). The specific mechanisms leading to therapy resistance in individuals remain unclear, prompting the implementation of various etiology-driven therapeutic strategies such as novel antiepileptic drug regimens, focal cortical resection and neurostimulation technologies (5, 6).

Surgical resection is considered the most effective amongst these therapeutic approaches (1, 7, 8). One state-of-the-art surgical technique, MRI-guided Laser Interstitial Thermotherapy (MRgLITT), traditionally used in the treatment of benign and malignant neoplasms, movement disorders, has more recently been employed in focal and generalized epilepsy (9). In the latter, MRgLITT is utilized primarily for palliative disconnected procedures—such as laser corpus callosotomy—targeting syndromes characterized by widespread electrical discharges and drop attacks that are not amenable to focal resection (9). While recent literature advocates for the use of rational polytherapy and ongoing evaluation of new antiepileptic drugs (AEDs) for drug-resistant epilepsy (10), the American Society for Stereotactic and Functional Neurosurgery (ASSFN) advocates for the use of MRgLITT in focal DRE (11).

MRgLITT is a minimally invasive procedure that has been associated with shorter hospital stays, reduced operative times, decreased blood loss, lower seizures and a decrease in their severity were observed, resulting in a lower recurrence rate and less severe episodes for the patient (12–17). Its usefulness has been demonstrated across various RE etiologies (18). However, available evidence lacks direct comparisons with open surgical approaches, and only weakly methodological and non-experimental study designs have been reported, without following any standardized guidelines. Hence, there is still considerable uncertainty regarding the actual impact of MRgLITT on DRE. The reason for conducting an umbrella review, rather than a systematic review *de novo*, lies in the need to synthesize existing evidence, which presents heterogeneous findings and methodologies. Furthermore, the added value of this study over previously published systematic reviews is its ability to offer an integrated view of the state of the art, evaluating the methodological robustness of these reviews using standardized tools (such as AMSTAR 2).

Despite the broad number of systematic reviews on the use of MRgLITT for DRE, included studies are dispersed throughout its different etiologies and do not comprehensively summarize its effects on DRE. Consequently, there is a significant absence of consensus and standardized recommendations for its clinical application. Therefore, this study aims to conduct a systematic overview of aggregated data from systematic reviews, with the objective of providing a descriptive evaluation of the evidence quality concerning the application of MRgLITT for drug-resistant epilepsy.

## 2 Materials and methods

### 2.1 Protocol and registration

This systematic review was reported following the Preferred Reporting Items for Systematic Reviews and Meta-Analyses (PRISMA) (19) guidelines. The protocol was previously registered in PROSPERO with the code: CRD42022339024 (20). See the PRISMA checklist as the Supplementary Table S1.

### 2.2 Literature search and study selection

We conducted searches in PubMed/Medline, Embase, Scopus, Web of Science, Cochrane Library and Google Scholar up to May 30th, 2024. The following terms were used for the search: “Drug-resistant epilepsy”, “Magnetic Resonance Imaging” and “Laser Therapy”. The complete search strategy is available in Supplementary Table S2. Our initial search was supplemented by including systematic reviews (SRs) cited in previous reviews and selecting those that met our eligibility criteria. Search strategy was independently applied to each database, and all retrieved records were processed with EndNote X9 software for duplicates removal. Subsequently, they were exported to Microsoft Excel for the classification of the thermal ablation intervention using special filters (9 groups were generated). Once classified, they were imported into EndNote X9 by “tab delimited” in txt format. Finally, they were exported in.XML format from EndNoteX9 to Mendeley, to transform the data into RIS files for Rayyan upload. Initially, each study was independently assessed by 2 authors (CQV, MCL) who used the eligibility criteria to determine their inclusion, by reviewing the title and abstract. Any disagreements were resolved through initial discussion between the two reviewers and a further arbitrator (FT) decision if no consensus was achieved. A similar approach was followed for full-text revision. SRs were included if they met the following criteria: (1) SRs (with or without metanalysis); (2) patients with DRE (according to ILAE Task Force 2009 consensus) (3); and (3) SRs that reported our prioritized outcomes: Seizure freedom (according to Engel or ILAE classification) at ≥6 months; perioperative or postoperative complications; and reoperation rates. We excluded literature reviews, animal studies, conference abstracts, posters, letters, and original primary studies.

### 2.3 Data extraction

Data extraction was independently carried out by four authors (FT, CQV, MCL, DSM) using a Microsoft Excel 2016 form which included the following information: author, publication year, country, language, study design, number of included participants; baseline characteristics such as gender (female or male), age, DRE etiology; intervention characteristics such as delivery platform (Visualase or Neuroblate), radiosurgical alternatives (comparators in the included studies); as well as, other methodological features like having performed a subgroup analysis, publication bias assessment or GRADE assessment.

### 2.4 Risk of bias assessment

The risk of bias and quality of the SRs were assessed using the Assessing the Methodological Quality of Systematic Reviews (AMSTAR)-2 tool (21), which is composed of 16 items. Most relevant domains (22) account for protocol registration prior to starting the SRs (item 2), adequacy of the literature search (item 4), exclusion reasons (item 7), risk of bias assessment (item 9), meta-analysis (item 11), interpretation of results (item 13), and assessment of publication bias (item 15). Its purpose is rating the overall confidence of assessed SRs in 4 categories (Critically low: More than one critical flaw with or without non-critical weaknesses; Low: One critical flaw with or without non-critical weaknesses; Moderate: More than one non-critical weakness; High: None or one non-critical weakness) based on some critical domains (items: 2, 4, 7, 9, 11, 13 and 15).

### 2.5 Outcomes

The primary outcome was effectiveness, measured by the seizure freedom rate (encompassing Engel Class I and ILAE Class I). The secondary outcome was safety, covering both perioperative complications (including intraoperative events such as intracranial or fiber-related hemorrhage, missed lesioning target, or inaccurate placement) and postoperative complications (such as neurological deficits [motor, visual field, or cognitive], optic neuritis, headache, mood disorders [anxiety, depression], and radiation necrosis), as well as the reoperation rate (defined as the need for subsequent ablations, whether due to surgical failure, planned staged treatment for complex lesions, or rescue therapy). All the pooled estimates were assessed at the last follow-up, as per each individual study.

### 2.6 Data synthesis

This systematic review primarily aimed to provide a narrative synthesis, describing the clinical and demographic characteristics of DRE cases retrieved from available literature and postoperative course after treatment with MRgLITT. We collected pooled estimates from SRs with or without metanalysis. Since SRs were being assessed, no meta-analysis, subgroup analysis, publication bias assessment, or sensitivity analysis was performed.

We performed a narrative description of our prioritized outcomes (seizure freedom, perioperative complications, and reoperation rate) by designing a bubble plot using R studio version 4.2.2, where each bubble represents one of the systematic reviews. Three bubble charts display information in the following dimensions: 1) The *x*-axis demonstrates each of the main outcomes (seizure freedom, perioperative or postoperative complications, and reoperation rate) reported by each study. 2) The *y*-axis demonstrates the AMSTAR-2 assessment score, and 3) the number of patients included in each SRs is translated into the bubble size and heatmap. Finally, the footnote shows the main RE etiologies identified on each SR.

### 2.7 Overlap assessment

To assess the degree of redundancy and overlap between the systematic reviews included, the GROOVE (Graphical Representation of Overlap for Overviews) tool was used (23). The analysis was based on the calculation of the Corrected Coverage Area (CCA), which adjusts the total overlap according to the number of unique studies and the number of reviews to avoid interpretation biases. Intersections where a primary study was included in a specific review were marked with a value of one. This structure made it possible not only to obtain an overall overlap value for the entire matrix, but also to assess the density of evidence and the interconnectivity between the different nodes of information collected.

### 2.8 Certainty of evidence assessment

As recommended by Cochrane (24), we assessed the certainty of the evidence (pooled estimates) from our included SRs in the quantitative synthesis. The grading of recommendation, assessment, development, and evaluation (GRADE) instrument was used. This critical appraisal was based on considerations such as study design, inconsistency, indirectness, imprecision, and publication bias for downgrading, as well as, large effect, confounding and dose-response relation for upgrading the certainty of evidence, as stated in the GRADE handbook (25). To account for SRs with overlapping papers, we filter out those studies repeated twice or more in different SRs, avoiding a decrease in the internal validity of our results due to overrepresentation of overlapped populations.

## 3 Results

### 3.1 Study selection

**Figure 1:**
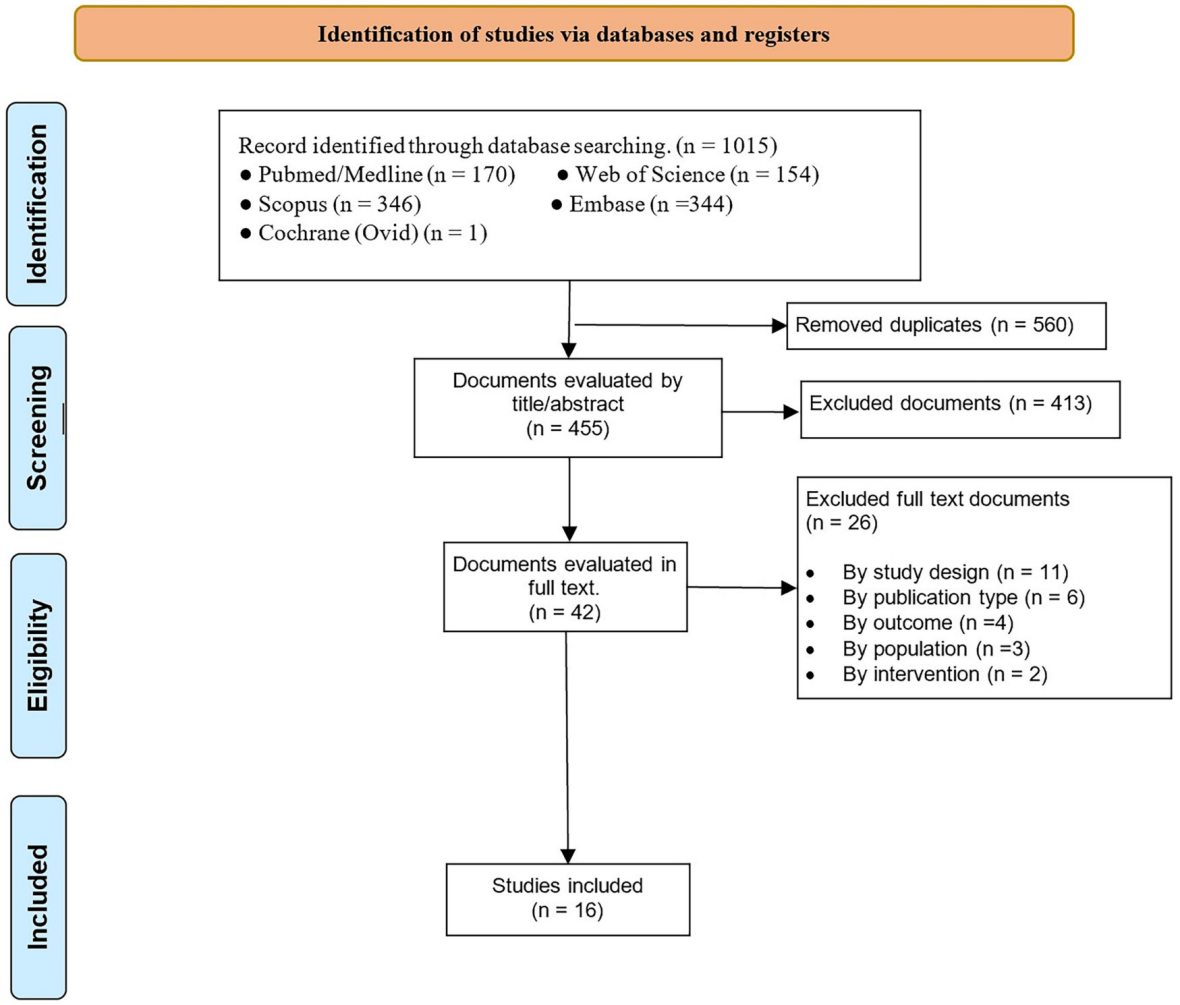
Selection process flowchart PRISMA 2020.

A total of 1,015 documents were initially identified. After removing duplicates and conducting the initial screening, followed by full-text selection process, 16 SRs were included based on the eligibility criteria (Figure 1). The list of reasons for exclusion can be found in Supplementary Table S3.

### 3.2 Study characteristics

Pooled estimates for our prioritized outcomes and covariates were derived from 16 SRs encompassing a total of 207 studies with 4,485 patients, of which at least 97 studies and 2,088 patients were non-overlapped. Most participants were male (ranging from 13.51% to 87.44%), with ages ranging from 0.5 to 74 years. The predominant etiologies targeted by the intervention were mesial temporal lobe epilepsy and hypothalamic hamartoma. The specific delivery platforms (e.g., Visualase, NeuroBlate) were detailed in 3 SRs (26–28). Six SRs (29–34) included other minimally invasive interventions (such as stereotactic radiosurgery, gamma-knife surgery, and stereo electroencephalography-guided radiofrequency thermocoagulation) as a comparators. All SRs included used the Engel classification to assess seizure freedom and described the most common perioperative and postoperative complications. Notably, the SRs exclusively incorporated observational studies, so the efficacy could not be measured. The characteristics of the SRs are summarized in Table 1.

**Table 1:** Characteristics of included systematic reviews evaluating the effectiveness of MRgLITT on refractory epilepsy.

Systematic review-ID	Number of included studies (participants)	Type of included studies	Male participants (%)	Age participants (SD)	Main etiology (%)	MRgLITT platform (%)	Comparator	MRgLITT seizure freedom (%)	MRgLITT complications (%)	MRgLITT reoperation rate (%)	Follow-up (months)	GRADE assessment (Overall quality of evidence)	AMSTAR-2 (Score)
Alomar et al. (37)	21 (836)	Retrospective and prospective cohorts	50.0%	40.8^a^ (5.12)	Temporal lobe epilepsy	NR	None	56% (95% CI: 52.4–59.5%)	19.8% (95% CI, 13.4–28.1%): Visual field deficits, intracranial hemorrhage, optic neuritis, headache, incisional pain, anxiety, depression, missed lesioning target.	NR	21.3^a^ (10.42)	NR	5.0/16 **Critically low**
Kohlhase et al. (31)	13 (554)	Single-arm observational studies	47%	43.1 (5.9)	Mesial temporal lobe epilepsy with hippocampal sclerosis (100%)	NR	RFA, ATL, sAHE	57%	Homonymous hemianopsia (1.91%), Intracranial hemorrhages (1.15%), subdural hematoma (0.38%)	NR	6–70	NR	8.0/16 **Critically low**
Marathe et al. (32)	11 (520)	Retrospective, prospective studies	NR	41.9	Mesial temporal lobe epilepsy (100%)	NR	ATL, trans-cortical and trans-sylvian SAH, SRS, RF-TC	59% (95% CI: 53.0–65.0%)	Visual field disfunction (7.5%), III and IV cranial nerve palsies (1.5%), postoperative hemorrhage (0.96%)	NR	12–42	NR	8.0/16 **Critically low**
Grewal et al. (34)	9 (249)	Retrospective cohorts	53.9%	40.9 (14.0)	Mesial temporal lobe epilepsy (99.2%)	NR	SRS, GKRS	50.0% (95% CI: 44–56%)	Visual Field deficits (5.2%) Hemorrhage (1.6%) Cognitive deficits (3.2%)	15%	7–70	Low	6.0/16 **Critically low**
Xue (17)	16 (269)	Retrospective and prospective cohorts, single-arm studies and case series	NR	7–69^b^	Mesial temporal lobe epilepsy (49.07%), temporal lobe epilepsy (21.19%)	NR	None	61% (95% CI, 0.54–0.68)	Postoperative complications 24% (95% CI, 0.16–0.32)	NR	7–51	NR	10.0/16 **Critically low**
Brotis et al. (28)	16 (575)	Case reports	NR	NR	NR	Visualase (100%)	None	54.7% (95%CI: 50.6–58.8%)	Persistent visual defect (0.2%)	NR	13^a^	Very low	12.0/16 **Low**
Rizzi et al. (30)	7 (130)	Retrospective cohorts, case series and a case report.	NR	0.5–68.9^b^	Hypothalamic hamartomas	NR	Open microsurgery Endoscopic surgery Radiosurgery RF-TC	75.86% (95% CI, 56.46–89.70)	Complications (88.46%), Neurological complications: 6.96% (95% CI: 3.05–13.25),	NR	12–27^b^	NR	8.0/16 **Critically low**
Wang et al. (33)	16 (414)	Retrospective, prospective studies	87.44%	0.4–74^b^	Temporal lobe epilepsy (64.25%), Hypothalamic hamartoma (20.05%)	NR	SEEG-RF-TC	65% (95% CI 56%–74%)	Visual field deficit (2.17%), neurological deficit (1.69%), inaccurate fiber placement or device malfunction (0.97%)	NR	6–42.9	NR	8.0/16 **Critically low**
Ilse (35)	27 (184)	Retrospective cohort, Prospective cohort and Case reports	NR	NR	Temporal lobe epilepsy (57.1%) Hypothalamic hamartomas (15.2%)	Visualase (88.0%) Neuroblate (9.8%)	None	73.0%	Radiation necrosis Spinal ependymoma Catheter misplacements	NR	1–48	NR	2.0/16 **Critically low**
Chen et al. (26)	46 (450)	Retrospective cohort and case series	50.4%	29.5 (18.1)	MTS/A (33.7%), HH (16.3%), MCD (14.4%), CCM (8.2%), TSC (5.9%)	Visualase [Medtronic] (95.5%) Neuroblate [Monteris Medical] (4.5%)	None	57.8%	Operative complications (8.5%), Postoperative neurological deficits (15.1%)	14.5%	22 (13.7)	Low	8.5/16 **Critically low**
Li et al. (29)	6 (37)	Retrospective cohorts	13.51%	8–20^b^	Focal cortical dysplasia	NR	SEEG-RF-TC	59% (95% CI: 44%–75%)	89% (95% CI: 77%–100%), Postoperative complications (32.4%)	NR	1–35^b^	NR	8.0/16 **Critically low**
Kerezoudis et al. (38)	2 (6)	Retrospective cohort and case series	50.0%	34.8	Cavernoma (50%)	NR	None	66.7%	Intracranial hemorrhage (50%)	NR	19.5	NR	2.0/16 **Critically low**
Awad et al. (27)	10 (57)	Retrospective cohorts	62.5%	25.6^a^	NR	Visualase (96.6%) NeuroBlate (3.4%)	None	21.1%	Fiber-related hemorrhage (8.6%) Inaccurate placement (6.9%) Transient hemiparesis (5.2%)	NR	3.6–39.6	NR	6.5/16 **Critically low**
Barot et al. (18)	28 (559)	Retrospective and prospective cohorts	23.0%	Adults: 35.0–60.0^b^ Pediatric: 6.6–12.8	Mesial temporal lobe epilepsy (45.1%) Hypothalamic hamartoma (16.8%)	NR	None	56.0% (95% CI: 52.0–60.0%)	Visual field deficits (5.4%) Motor deficits (4.8%) Intracranial hemorrhage (2.3%)	9%	7–42.9	NR	14.0/16 **Low**
Hect et al. (36)	16 (85)	Case reports, case series, and a case-control study	67.35%	20.78 (1–52)^b^	Lennox Gastaut Syndrome (58.44%)	NR	None	18.87%	Operative complications (12.94%), Other Complications (27.06%)	7.06%	17.37 (0.5–48)	Very low	5.5/16 **Critically low**
Badger et al. (16)	6 (13)	Case series	NR	12.4–32.5^b^	Atonic seizures, complex partial seizures, tonic/atonic seizures, behavioral arrest/tonic seizures, generalized tonic-clonic seizures, absence seizures	NR	None	45.45%	24% (IC 95%: 16–32): Transient increase in seizure activity, Transient hypersomnia, Inaccurate placement, Transient SMA syndrome Catheter associated hemorrhage	NR	5–39.7	NR	4.0/16 **Critically low**

a Median.

b Range.

MRgLITT, Magnetic resonance-guided laser interstitial thermal therapy; SRS, Stereotactic radiosurgery; GKRS, GammaKnife radiosurgery; RFA, Radiofrequency ablation; ATL, Anterior temporal lobe resection; SAH, Selective amygdalohippocampectomy; SEEG-RF-TC, Stereo electroencephalography-guided radiofrequency thermocoagulation; SMA, Suplemmentary motor area; NR, No reported; MTS/A, Mesial Temporal Sclerosis/Atrophy; HH, Hypothalamic Hamartoma; MCD, Malformations of Cortical Development; CCM, Cerebral Cavernous malformation; TSC, Tuberous Sclerosis complex.

### 3.3 Subgroup evaluation

A synthesis of the data was performed by grouping the studies according to the anatomical location of the epileptogenic zone and the type of ablative procedure. The largest subgroup corresponded to temporal lobe epilepsy (TLE), represented by the studies by Alomar et al. (37), Kohlhase et al. (31), Marathe et al. (32), Grewal et al. (34), Xue et al. (17), Brotis et al. (28), Wang et al. (33) and Ilse et al. (35). In this group, the seizure-free rate remained consistent ranging from 50.0% to 61%. However, the safety profile for this location was notable for a significant incidence of visual field deficits (specifically homonymous hemianopsia), reported between 1.91% and 19.8% due to the involvement of optic radiation. Likewise, a reoperation rate of up to 15% was recorded, where the predominant secondary procedure was conversion to traditional open surgery, such as anterior temporal lobectomy, after the failure of the initial thermocoagulation.

On the other hand, procedures targeting deep structure lesions and hamartomas, analyzed in the studies by Rizzi et al. (30), Wang et al. (33) and Ilse et al. (35) (HH cases), showed the highest success rates in the review, reaching between 73.0% and 75.8% seizure freedom. Although permanent neurological complications were low (6.9%), high transient morbidity of up to 88.4% was observed in some reports, characterized by electrolyte imbalances, hyperphagia, and diabetes insipidus, resulting from the manipulation of autonomic and hypothalamic structures. In these cases, reoperation mainly consisted of repeating LITT ablation to complete the lesion volume in large hamartomas that did not respond to primary treatment.

In the subgroup of Cortical Developmental Malformations and Vascular Lesions, which includes reports on focal cortical dysplasia and cavernomas by Chen et al. (26), Li et al. (29), Kerezoudis et al. (38) and Awad et al. (27), efficacy results were more variable, ranging from 21.1% to 66.7%. This variability was associated with a notable risk of postoperative neurological deficits (15.1%) and hemorrhages related to the laser fiber trajectory, reaching up to a 50% hemorrhagic risk in the specific case of cavernomas (37). The reoperation rate in this group was 14.5%, mainly oriented towards open microsurgical lesionectomy to ensure total removal of the residual epileptogenic zone.

Finally, Disconnection Procedures, such as laser callosotomy evaluated by Barot et al. (18), Hect et al. (36) and Badger et al. (16), had a success rate ranging from 18.87% to 56.0%, reflected on the palliation of drop seizures. The complications reported were unique to this technique, including supplementary motor area syndrome and transient hypersomnia. Reoperation rates ranged from 7.06% to 9%, consisting of extension of the callosotomy (from partial to total) or, in refractory cases, the implementation of neuromodulation therapies such as vagus nerve stimulation (VNS) as a rescue strategy.

### 3.4 Risk of bias assessment

We assessed the risk of bias of the 16 SRs. According to critical item assessment in AMSTAR-2, 14 SRs (16, 17, 26, 27, 29–38) obtained a “critically low” quality of evidence due to lack of a review protocol, non-comprehensive search strategy, absence of an appropriate risk of bias and publication bias assessment, and of a quantitative synthesis through meta-analysis. Five SRs (28, 33, 35, 36, 38) showed no statement regarding funding nor conflicts of interest. The overall individual score of the SRs ranged from 2/16 to 14/16. Ilse et al. (35) obtained the lowest score: 2, while Barot et al. (18) obtained the highest one: 14 (Supplementary Figure S1). The review with high quality evidence analyzed 28 studies (559 patients with most adults) evaluating the effectiveness of MRgLITT in DRE and found an overall prevalence of Engel class I of 56% (95% CI: 0.52% to 0.60%) and of postoperative adverse events of 19% (95% CI: 0.14% to 0.25%), highlighting visual field deficit. They also found a higher rate of seizure freedom in DRE caused by hypothalamic hamartomas (67%), which would indicate a greater usefulness of MRgLITT in this group.

### 3.5 Outcomes

**Figure 2:**
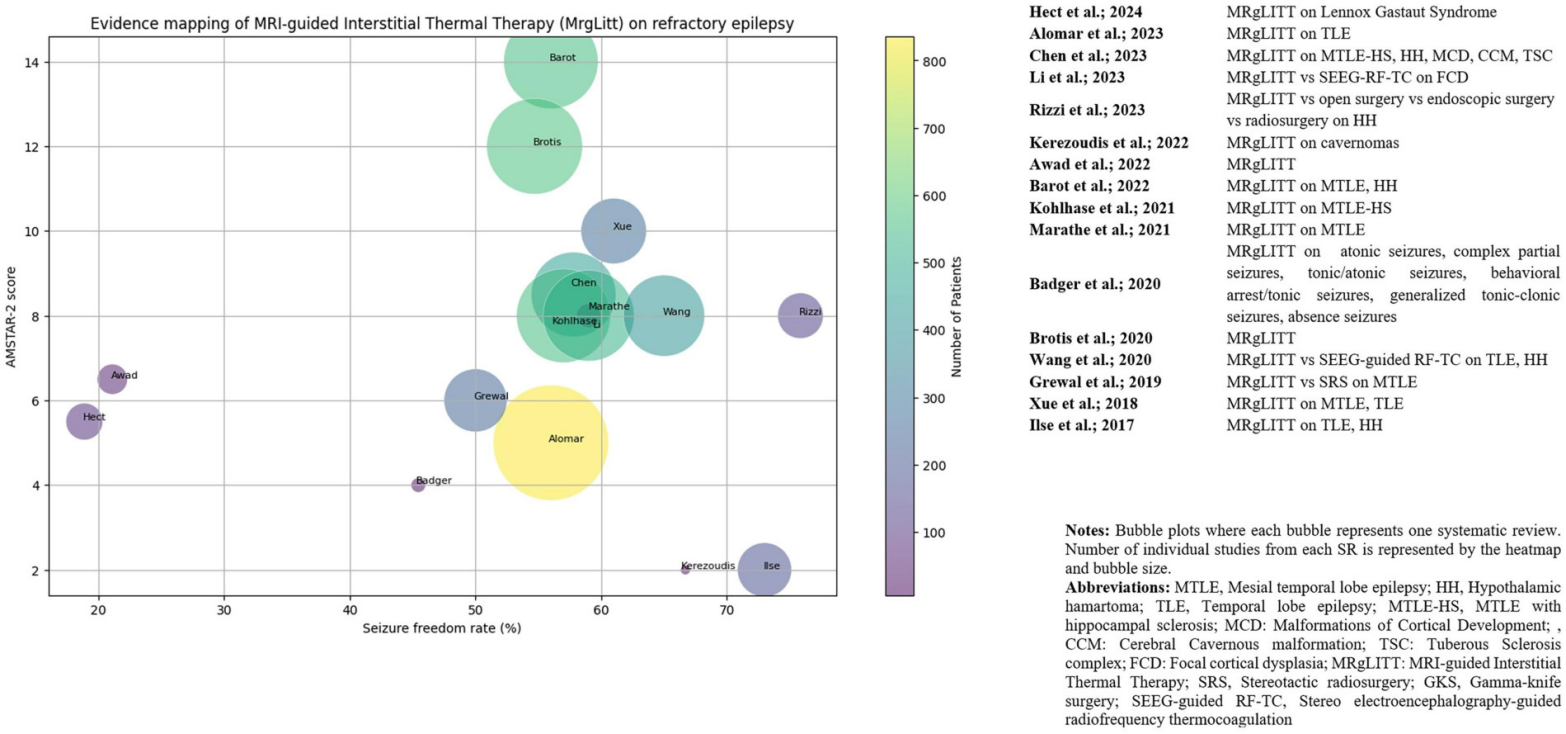
Evidence mapping-seizure freedom rate.

**Figure 3:**
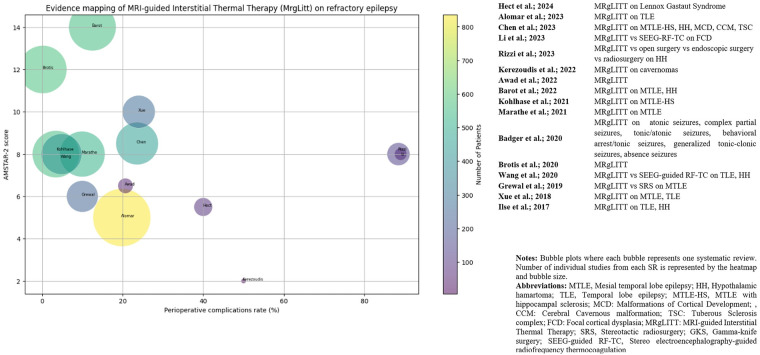
Evidence mapping-perioperative complications rate.

**Figure 4:**
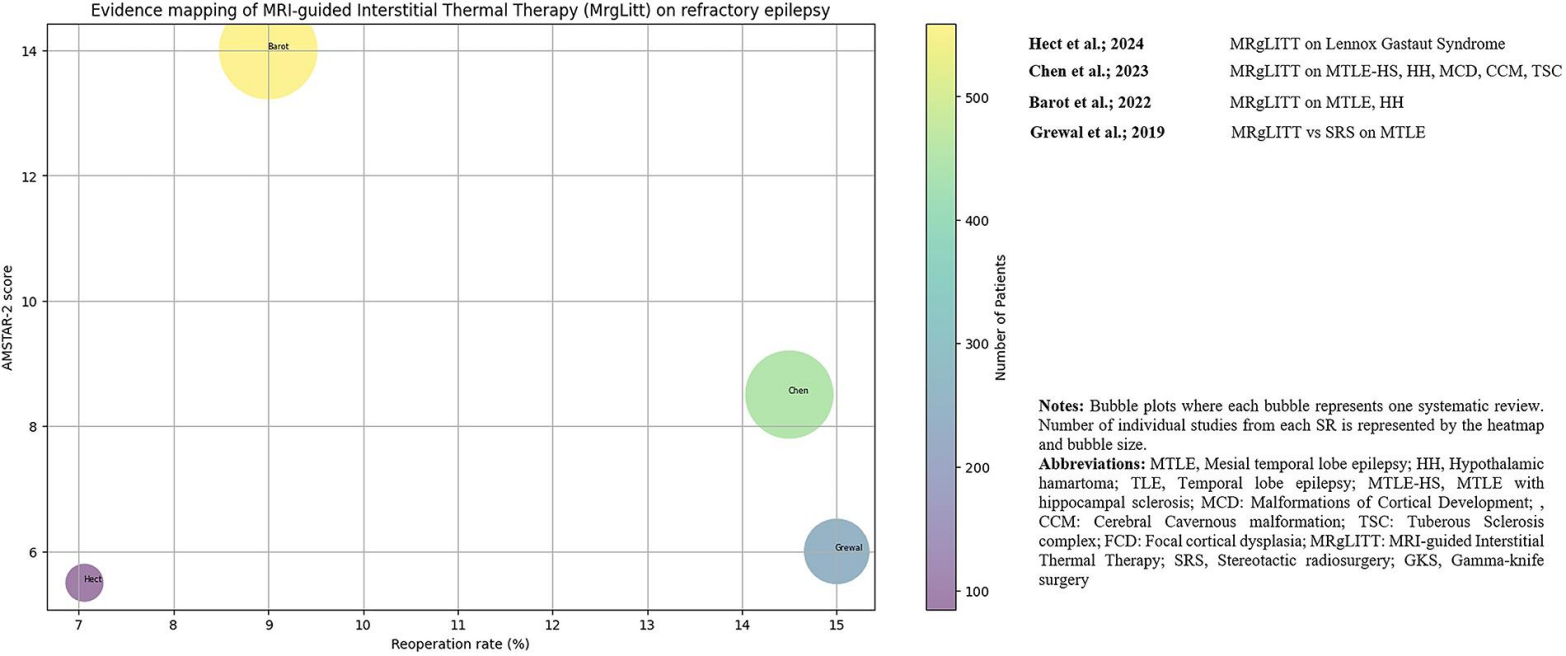
Evidence mapping-reoperation rate.

We represented the seizure freedom, perioperative or postoperative complication, and reoperation rate outcomes on the bubble plot (Figures 2–4).

Regarding seizure freedom rate, Rizzi et al. (30) reported the highest effectiveness (75.86%), while Hect et al. (36), the lowest (18.87%). Nine SRs (17, 18, 28–30, 32–34, 37) ran a meta-analysis and showed a pooled primary outcome. A total of 87 studies and 2,337 patients were included in the analysis. The pooled results showed a significant increase in seizure freedom, ranging from 50% (95% CI: 44–56) (34) to 75.86% (95% CI 56.46–89.70) (30). Perioperative complications rate was assessed through a follow-up duration ranging from 7 days to 70 months. Most commonly reported complications were visual field deficit, present in as much as 7.5% of cases (32), and fiber-related intracranial hemorrhage (8.6%), principally reported by Awad et al. (27). The SR presented by Badger et al. (16) reported the highest pooled prevalence of complications 24% (CI 95%: 16–32), such as a transient increase in seizure activity, transient hypersomnia, inaccurate placement, and catheter associated hemorrhage. Other identified complications such as cognitive deficit, device-related malfunction, cranial nerve deficits, edema, and hydrocephalus. Finally, reoperation rate was only assessed in 4 SRs (18, 26, 34, 36), varying from a minimum of 7.06% to a maximum rate of 15% (18, 34) (Table 1).

### 3.6 Overlap assessment

The overlap analysis using the GROOVE tool recorded a total of 249 citations included in the evidence matrix. The CCA calculated for the entire matrix was 9.29%, which is interpreted as moderate overlap. This result is derived from a gross coverage area of 14.96%, and since no structural zeros affecting the chronology or logic of inclusion were identified, the adjusted value remained at 9.29% (Supplementary Figure S2).

### 3.7 Summary of evidence according to GRADE

For the certainty of evidence analysis, we excluded reviews (16, 35) whose majority of studies were cases series or case reports due to the absence of pooled estimates reporting (Table 2). Regarding seizure freedom rate, we started the certainty of evidence assessment with a low value because all SRs included only observational studies. Therefore, the certainty was reduced by 3 points because most individual studies had a moderate risk of bias, as well as demonstrating a high indirectness by presenting different comparators, and a wide confidence interval (CI). The certainty for perioperative complications similarly started with a “low” grading. Then, it was reduced by 2 points due to high indirectness stemming from different comparators, and a wide CI. Overall certainty was rated as very low. Reoperation rate obtained a very low certainty, since the only review (18) reporting it had a high heterogeneity (I2 > 50%) and a very wide CI.

**Table 2:** GRADE summary of findings.

Outcomes	No of participants	No of included studies (Retrospective and prospective studies)	Effect (95% CI)	Certainty of evidence
Seizure freedom	1987	102	From 18.9 to 75.9	⊕◯◯◯^a^^,^^b^^,^^c^
				Very low
Perioperative and postoperative complications	2,141	75	From 7 (4–11) to 20 (14–26)	⊕◯◯◯^b^^,^^c^
				Very low
Reoperation rate	675	38	11.4 (7.06–15)	⊕⊕◯◯^c^^,^^d^^,^^e^
				Very low

Effect estimates are reported in percentages per 100 treated patients from a single arm.

a Moderate risk of bias of individual studies (>25%).

b High indirectness by presenting different comparators.

c High inaccuracy due to a large IC.

d The study shows a moderate heterogeneity (*I*^2^ > 50%).

e High risk of bias of individual studies (>25%).

## 4 Discussion

### 4.1 Main findings

This overview highlights the features of MRgLITT by summarizing all published data of seizure-freedom, perioperative or postoperative complication and reoperation rates, which could not be addressed in a single SR. Data were presented in reader-friendly charts (39) and evidence from sixteen SRs, including at least 97 non-overlapping studies conducted between 2012 and 2024 and involving 2088 participants, was retrieved.

### 4.2 Seizure freedom, complication rate and reoperation rate

MRgLITT demonstrated a variable seizure freedom rate (18.87%–75.86%) in patients with DRE, comparable to open surgical approaches. Open surgery is usually considered a last resource due to its challenging postoperative recovery course (40). However, MRgLITT exhibits a seven-fold higher seizure freedom rate compared to medical treatment at one-year follow-up (41, 42). The wide range of reported seizure freedom rates may be influenced by publication bias, stemming from one outlier study (27), which assessed a less common procedure (corpus callosotomy) with a distinct success rate not directly comparable to other DRE procedures such as lesionectomy or lobectomy. A more conservative estimate of seizure freedom rate, around 50%, is suggested by a systematic review conducted by Barot et al. (18), which had a more substantial sample size and higher methodological quality.

Perioperative complications rates varied from 0.2 to 19.8%, including symptomatic cerebral edema, worsening neurologic deficit, and intracranial hemorrhage (43). The mitigation of adverse effects in MRgLITT may be attributed to its MR-guided thermographic equipment, which accurately predicts damage and minimizes complications (44). Visual field defects were the most frequent complication, consistently reported as a transient, non-disabling neurological injury caused by initial edema surrounding the ablation site (14, 45, 46). Open surgery reports a comparable frequency of complications (5.1%) (47), primarily associated with procedural injuries to surrounding tissue such as cerebrospinal fluid leaks, aseptic meningitis and intracranial hematomas, necessitating prolonged inpatient postoperative care.

Approximately 15% of patients require a second ablation (34), positioning MRgLITT as a rescue treatment after surgical or radiotherapy failures (48). However, evidence on the repetition of sessions specifically in drug-resistant epilepsy is still limited.

Subgroup analysis reveals that the clinical utility of MRgLITT depends closely on the anatomical location and specific nature of the DRE. The technique exhibits its maximum potential in deep focal lesions, particularly in hypothalamic hamartomas (29, 32, 34), where it achieved the highest seizure-free rates (up to 75.8%). In these cases, MRgLITT overcomes the high risks of open surgery thanks to its assisted thermal precision; however, the high rate of transient hypothalamic dysfunction (88.4%) and the occasional need for re-ablation to complete the lesion volume during surgical planning must be considered. In contrast, results in ELTM and focal malformations (16, 25–28, 30–33, 36) show consistent efficacy (50%–61%) but critically depend on precise focus delineation. Our findings highlight that in MLTE, the risk of visual field deficits (up to 19.8%) remains a primary concern due to the proximity of Meyer's loop, while in vascular malformations such as cavernomas (37), the risk of hemorrhage is a limiting factor. Finally, disconnection procedures, such as callosotomies (15, 17, 35), lower seizure-free rates (18.8%–56%) do not represent technical failure, but rather a shift in therapeutic goal toward palliative care. In this context, MRgLITT focuses on interrupting propagation networks to mitigate catastrophic drop attacks, often requiring subsequent extension of the disconnection or supplementation with VNS. These differences imply that patient selection should not be based solely on drug resistance, but on an anatomical-functional analysis where LITT is established as the technique of choice for lesions that are difficult to access or that require the protection of adjacent eloquent tissue.

### 4.3 Certainty of evidence assessment

The overall certainty of evidence was deemed very low, primarily due to downgrades related to high confidence interval values and indirectness stemming from various comparators. Additionally, inconsistency was rated as “moderate” due to one study exhibiting a *I*^2^ value higher than 50% (18). The non-experimental nature of all the included SRs further introduces a significant risk of bias and confounding. To the extent of our knowledge, the lack of high-quality evidence precluded the development of guidelines in this field. Furthermore, only four SRs (18, 26, 34, 36) assessed the reoperation rate as an outcome, highlighting a gap in the current literature. Quality assessments were conducted in nine systematic reviews (17, 18, 26, 28, 30–34), with GRADE assessments performed in four SRs (26, 28, 34, 36), ensuring rigorous reporting according to current guidelines. Future SRs adhering to the criteria outlined in the Cochrane handbook for SRs developers (24) are needed to advance clinical research in this field.

The large number of reviews with “critically low” quality according to AMSTAR-2 suggests that the combined results should be interpreted with caution. However, the consistency between the findings of the limited reviews and the only high-quality review (Barot et al. 2022) lends some clinical validity to the reported success rates, especially in subgroups such as hypothalamic hamartomas. Ultimately, although the clinical trend is favorable, the methodological fragility of the current literature prevents definitive recommendations from being made without a substantial improvement in the standard of future research. Furthermore, the overlap findings demonstrate that, although there is a common base of frequently cited primary studies, the included reviews maintain a significant proportion of independent sources or diverse approaches, justifying the performance of this global synthesis to unify the scattered evidence.

### 4.4 Paradigm of MRgLITT for RE

The clinical utility of this intervention relies on the precise thermal ablation of epileptogenic tissue in patients ineligible for open surgery (49). This targets small focal epileptogenic zones such as mesial temporal lobe epilepsy, hypothalamic hamartomas and periventricular nodular heterotopia (50). However, its applicability is limited in cases of unclear epileptogenic foci, hemorrhagic conditions and healthcare facilities lacking this technology (11). Moreover, the intervention's costs increase significantly when not reimbursed by the health system or covered by insurance (12, 51). These factors contribute to its predominant adoption in North American and European countries. Given the identification of only low-quality systematic reviews, developing guideline recommendations necessitates addressing their primary shortcomings. This entails conducting primary experimental studies, specifically randomized or non-randomized clinical trials with adequate sample sizes focusing on specific DRE etiologies and comparative interventions. Additionally, evaluating the reoperation rate as an independent outcome could serve as an alternative measure of effectiveness compared to seizure freedom rates.

Despite this, the marked clinical heterogeneity, which covers an age range from 4 months to 74 years and etiologies as diverse as hypothalamic hamartomas and temporal sclerosis, severely limits the generalizability of our findings. This disparity in populations, coupled with variability in technological platforms and surgical protocols, is the main obstacle to the creation of definitive clinical practice guidelines. Without standardized criteria, the results should be considered promising trends rather than universal recommendations.

### 4.5 Limitations and strengths

This overview highlights several limitations. None of the SRs employed an experimental design, thus only reflecting the effectiveness through seizure freedom rates, rather than the efficacy. Most estimates are derived from critically low-quality reviews, which means that our findings must be interpreted with extreme caution, as methodological deficiencies in the underlying reviews could introduce biases that compromise the validity of conclusions about the effectiveness of MRgLITT. Furthermore, the potential presence of publication bias could not be assessed due to the absence of proper statistical analysis. Conducting a sensitivity analysis by removing outlier studies might offer a clearer perspective on the actual effectiveness of MRgLITT for DRE. However, the limited number of meta-analyses per outcome precludes conducting a meta-meta-analysis. Therefore, despite addressing a state-of-the-art minimally invasive intervention, a comprehensive analysis of all available systematic reviews would facilitate summarizing the peak of evidence-based medicine. Given the narrative nature of this overview, statistical analyses were not feasible to adjust for potential confounders, thereby limiting its knowledge contribution to the clinical applicability of this technique. Nevertheless, the initial search was unrestricted, mitigating the risk of publication bias, and results were interpreted considering the internal validity of the findings.

### 4.6 Conclusions and recommendations

This overview provides comprehensive evidence on the application of MRgLITT for drug-resistant epilepsy. The evidence indicates a seizure freedom rate ranging from 18.87% to 75.86% with certainty levels ranging from “critically low” to “low”. While these findings are promising, further primary and secondary studies are essential to establish definitive conclusions and assess the potential for future clinical applications or recommendations.

## Data Availability

The original contributions presented in the study are included in the Supplementary material, further inquiries can be directed to the corresponding author.
