# Supplementary material for: New insights from MRI-guided laser interstitial thermal therapy for refractory epilepsy: a state-of-the-art overview
Source: Front Surg. 2026 May 29;13:1642054. doi: 10.3389/fsurg.2026.1642054 (PMC13260251; doi:10.3389/fsurg.2026.1642054)
Supplement: Supplementary file 1 [file Supplementaryfile1.docx]

Supplementary Material

# Supplementary table 1. PRISMA 2020 Checklist

| **Section and Topic** | **Item #** | **Checklist item** | **Reported (Yes/No)** |
| --- | --- | --- | --- |
| **TITLE** | | |  |
| Title | 1 | Identify the report as a systematic review. | Yes |
| **BACKGROUND** | | |  |
| Objectives | 2 | Provide an explicit statement of the main objective(s) or question(s) the review addresses. | Yes |
| **METHODS** | | |  |
| Eligibility criteria | 3 | Specify the inclusion and exclusion criteria for the review. | Yes |
| Information sources | 4 | Specify the information sources (e.g. databases, registers) used to identify studies and the date when each was last searched. | Yes |
| Risk of bias | 5 | Specify the methods used to assess risk of bias in the included studies. | Yes |
| Synthesis of results | 6 | Specify the methods used to present and synthesise results. | Yes |
| **RESULTS** | | |  |
| Included studies | 7 | Give the total number of included studies and participants and summarise relevant characteristics of studies. | Yes |
| Synthesis of results | 8 | Present results for main outcomes, preferably indicating the number of included studies and participants for each. If meta-analysis was done, report the summary estimate and confidence/credible interval. If comparing groups, indicate the direction of the effect (i.e. which group is favoured). | Yes |
| **DISCUSSION** | | |  |
| Limitations of evidence | 9 | Provide a brief summary of the limitations of the evidence included in the review (e.g. study risk of bias, inconsistency and imprecision). | Yes |
| Interpretation | 10 | Provide a general interpretation of the results and important implications. | Yes |
| **OTHER** | | |  |
| Funding | 11 | Specify the primary source of funding for the review. | Yes |
| Registration | 12 | Provide the register name and registration number. | Yes |

| **Section and Topic** | **Item #** | **Checklist item** | **Location where item is reported** |
| --- | --- | --- | --- |
| **TITLE** | | |  |
| Title | 1 | Identify the report as a systematic review. | Title section |
| **ABSTRACT** | | |  |
| Abstract | 2 | See the PRISMA 2020 for Abstracts checklist. | Based on PRISMA 2020 for Abstracts checklist |
| **INTRODUCTION** | | |  |
| Rationale | 3 | Describe the rationale for the review in the context of existing knowledge. | Introduction section |
| Objectives | 4 | Provide an explicit statement of the objective(s) or question(s) the review addresses. | Introduction section |
| **METHODS** | | |  |
| Eligibility criteria | 5 | Specify the inclusion and exclusion criteria for the review and how studies were grouped for the syntheses. | Subsection 2.2 “Literature search and study selection” |
| Information sources | 6 | Specify all databases, registers, websites, organisations, reference lists and other sources searched or consulted to identify studies. Specify the date when each source was last searched or consulted. | Figure 1 PRISMA 2020 |
| Search strategy | 7 | Present the full search strategies for all databases, registers and websites, including any filters and limits used. | Subsection 2.2 “Literature search and study selection” |
| Selection process | 8 | Specify the methods used to decide whether a study met the inclusion criteria of the review, including how many reviewers screened each record and each report retrieved, whether they worked independently, and if applicable, details of automation tools used in the process. | Subsection 2.2 “Literature search and study selection” |
| Data collection process | 9 | Specify the methods used to collect data from reports, including how many reviewers collected data from each report, whether they worked independently, any processes for obtaining or confirming data from study investigators, and if applicable, details of automation tools used in the process. | Subsection 2.3 “Data extraction” |
| Data items | 10a | List and define all outcomes for which data were sought. Specify whether all results that were compatible with each outcome domain in each study were sought (e.g. for all measures, time points, analyses), and if not, the methods used to decide which results to collect. | Subsection 2.5 “Outcomes” |
|  | 10b | List and define all other variables for which data were sought (e.g. participant and intervention characteristics, funding sources). Describe any assumptions made about any missing or unclear information. | Subsection 2.5 “Outcomes” |
| Study risk of bias assessment | 11 | Specify the methods used to assess risk of bias in the included studies, including details of the tool(s) used, how many reviewers assessed each study and whether they worked independently, and if applicable, details of automation tools used in the process. | Subsection 2.4 “Risk of bias assessment” |
| Effect measures | 12 | Specify for each outcome the effect measure(s) (e.g. risk ratio, mean difference) used in the synthesis or presentation of results. | Subsection 2.6 “Data synthesis” |
| Synthesis methods | 13a | Describe the processes used to decide which studies were eligible for each synthesis (e.g. tabulating the study intervention characteristics and comparing against the planned groups for each synthesis (item #5)). | Subsection 2.6 “Data synthesis” |
|  | 13b | Describe any methods required to prepare the data for presentation or synthesis, such as handling of missing summary statistics, or data conversions. | Subsection 2.6 “Data synthesis” |
|  | 13c | Describe any methods used to tabulate or visually display results of individual studies and syntheses. | Subsection 2.6 “Data synthesis” |
|  | 13d | Describe any methods used to synthesize results and provide a rationale for the choice(s). If meta-analysis was performed, describe the model(s), method(s) to identify the presence and extent of statistical heterogeneity, and software package(s) used. | Subsection 2.6 “Data synthesis” |
|  | 13e | Describe any methods used to explore possible causes of heterogeneity among study results (e.g. subgroup analysis, meta-regression). | Subsection 2.6 “Data synthesis” |
|  | 13f | Describe any sensitivity analyses conducted to assess robustness of the synthesized results. | Subsection 2.7 “Overlap Assessment” |
| Reporting bias assessment | 14 | Describe any methods used to assess risk of bias due to missing results in a synthesis (arising from reporting biases). | Not applicable |
| Certainty assessment | 15 | Describe any methods used to assess certainty (or confidence) in the body of evidence for an outcome. | Subsection 2.8 “Certainty of evidence assessment” |
| **RESULTS** | | |  |
| Study selection | 16a | Describe the results of the search and selection process, from the number of records identified in the search to the number of studies included in the review, ideally using a flow diagram. | Subsection 3.1 “Study selection” |
|  | 16b | Cite studies that might appear to meet the inclusion criteria, but which were excluded, and explain why they were excluded. | Subsection 3.1 “Study selection” |
| Study characteristics | 17 | Cite each included study and present its characteristics. | Subsection 3.2 “Study characteristics” |
| Risk of bias in studies | 18 | Present assessments of risk of bias for each included study. | Subsection 3.3 “Risk of bias assessment” |
| Results of individual studies | 19 | For all outcomes, present, for each study: (a) summary statistics for each group (where appropriate) and (b) an effect estimate and its precision (e.g. confidence/credible interval), ideally using structured tables or plots. | Table 1 |
| Results of syntheses | 20a | For each synthesis, briefly summarise the characteristics and risk of bias among contributing studies. | Subsection 3.3 “Risk of bias assessment” |
|  | 20b | Present results of all statistical syntheses conducted. If meta-analysis was done, present for each the summary estimate and its precision (e.g. confidence/credible interval) and measures of statistical heterogeneity. If comparing groups, describe the direction of the effect. | Subgroup evaluation |
|  | 20c | Present results of all investigations of possible causes of heterogeneity among study results. | Not applicable |
|  | 20d | Present results of all sensitivity analyses conducted to assess the robustness of the synthesized results. | Not applicable |
| Reporting biases | 21 | Present assessments of risk of bias due to missing results (arising from reporting biases) for each synthesis assessed. | Not applicable |
| Certainty of evidence | 22 | Present assessments of certainty (or confidence) in the body of evidence for each outcome assessed. | Subsection 3.6 “Summary of evidence according to GRADE” |
| **DISCUSSION** | | |  |
| Discussion | 23a | Provide a general interpretation of the results in the context of other evidence. | Subsection 4.1 “Main Findings” |
|  | 23b | Discuss any limitations of the evidence included in the review. | Subsection 4.5 “Limitations and Strengths” |
|  | 23c | Discuss any limitations of the review processes used. | Subsection 4.5 “Limitations and Strengths” |
|  | 23d | Discuss implications of the results for practice, policy, and future research. | Subsection 4.4 “Paradigm of MRgLITT for RE” |
| **OTHER INFORMATION** | | |  |
| Registration and protocol | 24a | Provide registration information for the review, including register name and registration number, or state that the review was not registered. | Subsection 2.1 “Protocol and registration” |
|  | 24b | Indicate where the review protocol can be accessed, or state that a protocol was not prepared. | Subsection 2.1 “Protocol and registration” |
|  | 24c | Describe and explain any amendments to information provided at registration or in the protocol. | Subsection 2.1 “Protocol and registration” |
| Support | 25 | Describe sources of financial or non-financial support for the review, and the role of the funders or sponsors in the review. | Section 7 “Funding” |
| Competing interests | 26 | Declare any competing interests of review authors. | Section 5 “Conflict of Interest” |
| Availability of data, code and other materials | 27 | Report which of the following are publicly available and where they can be found: template data collection forms; data extracted from included studies; data used for all analyses; analytic code; any other materials used in the review. | All information can be found in the manuscript and supplementary material. |
| From: Page MJ, McKenzie JE, Bossuyt PM, Boutron I, Hoffmann TC, Mulrow CD, et al. The PRISMA 2020 statement: an updated guideline for reporting systematic reviews. BMJ 2021;372:n71. doi: 10.1136/bmj.n71. For more information, visit: http://www.prisma-statement.org/ | | | |

# Supplementary table 2. Search strategy

| Database | Search strategy | Results |
| --- | --- | --- |
| Pubmed/Medline | #1 Epilepsy[Mh] OR Seizures[Mh] OR epilep*[tiab] OR seizure*[tiab] OR convulsion*[tiab] OR “comitial disease”[tiab] OR (falling[tiab] AND sickness[tiab])  #2 "Magnetic Resonance Imaging"[Mh] OR "Magnetic Resonance Imaging, Interventional"[Mh] OR "Magnetic Resonance"[tiab] OR “magnetization transfer”[tiab] OR “NMR imaging”[tiab] OR “MR Tomography”[tiab] OR “NMR Tomography”[tiab] OR MRI[tiab]  #3 ("Laser Therapy"[Mesh] OR "Lasers"[Mesh] OR laser*[tiab])  #4 (litt[TIAB] or MRgLITT[TIAB])  #5 ("Systematic Review"[Publication Type] OR "Systematic Reviews as Topic"[Mesh] OR "Meta-Analysis"[Publication Type] OR "Meta-Analysis as Topic"[Mesh] OR "Review*"[TIAB] OR "Meta-Analys*"[TIAB] OR “Meta Analys*”[TIAB] OR Metanalys*[TIAB] OR Metaanalys*[TIAB])  #6 (#1 AND ((#2 AND #3) OR #4)) AND #5 | 170 |
| Scopus | #1 TITLE-ABS-KEY(epilep* OR seizure* OR convulsion* OR “comitial disease”) OR TITLE-ABS-KEY(falling w/2 sickness)  #2 TITLE-ABS-KEY(“Magnetic Resonance” OR “magnetization transfer” OR “NMR imaging” OR “*MR Tomography” OR *MRI)  #3 TITLE-ABS-KEY(laser*)  #4 TITLE-ABS-KEY(*litt)  #5 TITLE-ABS-KEY(Review* OR "Meta-Analys*" OR “Meta Analys*” OR Metanalys* OR Metaanalys*)  #6 (#1 AND ((#2 AND #3) OR #4)) AND #5 | 346 |
| Web Of Science | #1 TS=(epilep* OR seizure* OR convulsion* OR “comitial disease”) OR TS=(falling NEAR/2 sickness)  #2 TS=(“Magnetic Resonance” OR “magnetization transfer” OR “NMR imaging” OR “*MR Tomography” OR *MRI)  #3 TS=(laser*)  #4 TS=(*litt)  #5 TS=(Review* OR "Meta-Analys*" OR “Meta Analys*” OR Metanalys* OR Metaanalys*)  #6 (#1 AND ((#2 AND #3) OR #4)) AND #5 | 154 |
| Embase | #1 ('epilepsy' OR 'seizure')/exp OR (epilep* OR seizure* OR convulsion* OR ‘comitial disease’):ti,ab,kw OR (falling NEAR/2 sickness):ti,ab,kw  #2 'nuclear magnetic resonance'/exp OR ('magnetic resonance’ OR 'magnetization transfer' OR 'nmr imaging' OR 'mr tomography' OR 'nmr tomography' OR mri):ti,ab,kw  #3 laser/exp OR laser*:ti,ab,kw  #4 (litt OR mrglitt):ti,ab,kw  #5 'systematic review (topic)'/exp OR 'meta analysis'/exp OR review*:ti,ab,kw OR 'meta-analys*':ti,ab,kw OR 'meta analys*':ti,ab,kw OR metanalys*:ti,ab,kw OR metaanalys*:ti,ab,kw OR review:it  #6 (#1 AND ((#2 AND #3) OR #4)) AND #5 | 344 |
| Cochrane (OvidSP) | #1 MeSH descriptor: [Epilepsy] explode all trees  #2 MeSH descriptor: [Seizures] explode all trees  #3 (epilep* OR seizure* OR convulsion* OR ‘comitial disease’):ti,ab,kw OR (falling NEAR/2 sickness):ti,ab,kw  #4 MeSH descriptor: [Magnetic Resonance Imaging] explode all trees  #5 MeSH descriptor: [Magnetic Resonance Imaging, Interventional] explode all trees  #6 ('magnetic resonance’ OR 'magnetization transfer' OR 'nmr imaging' OR 'mr tomography' OR 'nmr tomography' OR mri):ti,ab,kw  #7 MeSH descriptor: [Laser Therapy] explode all trees  #8 MeSH descriptor: [Lasers] explode all trees    #9 laser*:ti,ab,kw  #10 (litt OR mrglitt):ti,ab,kw    #11 (#1 OR #2 OR #3) AND (((#4 OR #5 OR #6) AND (#7 OR #8)) OR #10)  Filter: Cochrane reviews | 1 |

# Supplementary table 3. Excluded studies

| **Author-year** | **Title** | **Exclusion reasons** |
| --- | --- | --- |
| Brenner-2024 | Functional outcomes in MRI-guided laser interstitial therapy for temporal lobe epilepsy: a systematic review and meta-analysis | Wrong outcome because they did not evaluate the outcomes of seizure freedom, perioperative and postoperative complications, or reoperation rate. |
| Brown-2023 | The array of treatments available for hypothalamic hamartoma in the setting of intractable seizure: Current state of the evidence for the endoscopic disconnection (and resection), LITT, and radiotherapy | Wrong publication type because it is an abstract |
| Lee-2023 | Accuracy of robot-assisted stereotactic MRI-guided laser ablation in children with epilepsy | Wrong outcome because they evaluated factors associated with the accuracy of MRgLITT |
| Obaid-2023 | Predictors of outcomes after surgery for medically intractable insular epilepsy: A systematic review and individual participant data meta-analysis | Wrong outcome because they only evaluated the predictors of outcomes |
| Adin-2021 | Imaging of neuromodulation and surgical interventions for epilepsy | Wrong study design because it is a narrative review |
| Alomar-2021 | Neurocognitive outcome after laser interstitial ablation for temporal lobe epilepsy, a systematic review and meta-analysis | Wrong outcome because they did not evaluate the outcomes of seizure freedom, perioperative and postoperative complications, or reoperation rate. |
| Keator-2021 | Epilepsy surgery is viable treatment for Lennox Gastaut Syndrome | Wrong publication type because it is a narrative review that included other procedures different from MRgLITT |
| Wang-2021 | A systematic review of minimally invasive procedures for mesial temporal lobe epilepsy: Too minimal, too fast? | Wrong intervention because they included other procedures different from MRgLITT |
| Zeller-2021 | Current applications and safety profile of laser interstitial thermal therapy in the pediatric population: A systematic review of the literature | Wrong study design because it is a narrative review |
| Bastos-2020 | The use of laser interstitial thermal therapy in the treatment of brain metastases: a literature review | Wrong study design because it is a narrative review |
| Dorfer-2020 | Surgery for temporal lobe epilepsy | Wrong study design because it is a narrative review |
| Eichberg-2020 | Multiple iterations of magnetic resonance-guided laser interstitial thermal ablation of brain metastases: Single surgeon’s experience and review of literature | Wrong study design because it is a narrative review |
| Frassanito-2020 | Current trends in the management of subependymal giant cell astrocytomas in tuberous sclerosis | Wrong study design because it is a narrative review |
| Jimenez-2024 | Laser interstitial thermal therapy for cavernous malformations: a meta-analysis of individual patient-level data | Wrong population because they included populations that did not present drug-resistant epilepsy |
| Vale-2014 | LASER ABLATION THERAPY FOR THE MANAGEMENT OF MEDICALLY-RESISTANT EPILEPSY AFTER AGE 50 | Wrong publication type because it is an abstract |
| Youngerman-2020 | Magnetic resonance imaging-guided laser interstitial thermal therapy for epilepsy: Systematic review of technique, indications and outcomes | Wrong study design because it is a narrative review |
| Brown-2018 | Curative and palliative MRI-guided laser ablation for drug-resistant epilepsy | Wrong study design because it is a narrative review |
| Bourdillon-2018 | Stereo-electroencephalography-guided radiofrequency thermocoagulation in patients with focal epilepsy: A systematic review and meta-analysis | Wrong intervention because they included other procedures different from MRgLITT |
| Coyle-2017 | From resection to ablation: A review of resective surgical options for temporal lobe epilepsy and rationale for an ablation-based approach | Wrong study design because it is a narrative review |
| Du-2017 | Laser interstitial thermal therapy: A first line treatment for seizures due to hypothalamic hamartoma | Wrong study design because it is a narrative review |
| Lagman-2017 | Laser neurosurgery: A systematic analysis of magnetic resonance-guided interstitial thermal therapies | Wrong population because they included populations that did not present drug-resistant epilepsy |
| NICE-2020 | Interventional procedure overview of MRI-guided laser interstitial thermal therapy for drug-resistant epilepsy | Wrong publication type because it is a guideline |
| Jehi-2016 | Not all that glitters is gold: A guide to surgical trials in epilepsy | Wrong outcome because they compared different surgical methods and do not evaluate the outcomes of our umbrella review |
| Li-2024 | Postoperative seizure and memory outcome for temporal lobe epilepsy with different subtypes of hippocampal sclerosis: a systematic review and meta-analysis | Wrong publication type because it is an abstract |
| Hooper-2023 | Neurocognitive and psychosocial issues in adults with newly diagnosed epilepsy – a systematic review | Wrong publication type because it is an abstract |
| Choi-2014 | MRI-guided laser interstitial thermal therapy of intracranial tumours and epilepsy: State-of-the-art review and case study from pediatrics | Wrong study design because it is a narrative review |

# Supplementary Figure 1. A Measurement Tool to Assess systematic Reviews-AMSTAR 2

| **Study-ID** | **AMSTAR-2** | | | | | | | | | | | | | | | | |
| --- | --- | --- | --- | --- | --- | --- | --- | --- | --- | --- | --- | --- | --- | --- | --- | --- | --- |
|  | **Item 1** | **Item 2** | **Item 3** | **Item 4** | **Item 5** | **Item 6** | **Item 7** | **Item 8** | **Item 9** | **Item 10** | **Item 11** | **Item 12** | **Item 13** | **Item 14** | **Item 15** | **Item 16** | **Overall** |
| Hect et al.; 2024 |  |  |  |  |  |  |  |  |  |  |  |  |  |  |  |  | **CL** |
| Alomar et al.; 2023 |  |  |  |  |  |  |  |  |  |  |  |  |  |  |  |  | **CL** |
| Chen et al.; 2023 |  |  |  |  |  |  |  |  |  |  |  |  |  |  |  |  | **CL** |
| Li et al.; 2023 |  |  |  |  |  |  |  |  |  |  |  |  |  |  |  |  | **CL** |
| Rizzi et al.; 2023 |  |  |  |  |  |  |  |  |  |  |  |  |  |  |  |  | **CL** |
| Awad et al.; 2022 |  |  |  |  |  |  |  |  |  |  |  |  |  |  |  |  | **CL** |
| Barot et al.; 2022 |  |  |  |  |  |  |  |  |  |  |  |  |  |  |  |  | **L** |
| Kerezoudis et al.; 2022 |  |  |  |  |  |  |  |  |  |  |  |  |  |  |  |  | **CL** |
| Kollhase et al.; 2021 |  |  |  |  |  |  |  |  |  |  |  |  |  |  |  |  | **CL** |
| Marathe et al.; 2021 |  |  |  |  |  |  |  |  |  |  |  |  |  |  |  |  | **CL** |
| Badger et al.; 2020 |  |  |  |  |  |  |  |  |  |  |  |  |  |  |  |  | **CL** |
| Brotis et al.; 2020 |  |  |  |  |  |  |  |  |  |  |  |  |  |  |  |  | **L** |
| Wang et. al, 2020 |  |  |  |  |  |  |  |  |  |  |  |  |  |  |  |  | **CL** |
| Grewal et. al, 2019 |  |  |  |  |  |  |  |  |  |  |  |  |  |  |  |  | **CL** |
| Xue et. al, 2018 |  |  |  |  |  |  |  |  |  |  |  |  |  |  |  |  | **CL** |
| Ilse et. al, 2017 |  |  |  |  |  |  |  |  |  |  |  |  |  |  |  |  | **CL** |
|  |  |  |  |  |  |  |  |  |  |  |  |  |  |  |  |  |  |
|  |  |  |  |  |  |  |  |  |  |  | H: High | | | |  |  |  |
|  |  | Completely fulfills criteria | | | | | |  |  |  | M: Moderate | | | |  |  |  |
|  |  | Partially fulfills criteria | | | | |  |  |  |  | L: Low | | | |  |  |  |
|  |  | Does not fulfill criteria | | | | |  |  |  |  | CL: Critically low | | | |  |  |  |

| **Items** |
| --- |
| 1. Did the research questions and inclusion criteria for the review include the components of PICO? |
| 2. Did the report of the review contain an explicit statement that the review methods were established prior to the conduct of their review, and did the report justify any significant deviations from the protocol?***** |
| 3. Did the review authors explain their selection of the study designs for inclusion in the review? |
| 4. Did the review authors use a comprehensive literature search strategy?***** |
| 5. Did the review authors perform study selection in duplicate? |
| 6. Did the review authors perform data extraction in duplicate? |
| 7. Did the review authors provide a list of excluded studies and justify the exclusions?***** |
| 8. Did the review authors describe the included studies in adequate detail? |
| 9. Did the review authors use a satisfactory technique for assessing the risk of bias (RoB) in individual studies that were included in the review?***** |
| 10. Did the review authors report on the sources of funding for the studies included in the review? |
| 11. If meta-analysis was performed, did the review authors use appropriate methods for statistical combination of results?***** |
| 12. If meta-analysis was performed, did the review authors assess the potential impact of RoB in individual studies on the results of the metanalysis or other evidence synthesis? |
| 13. Did the review authors account for RoB in individual studies when interpreting/discussing the results of the review?***** |
| 14. Did the review authors provide a satisfactory explanation for, and discussion of, any heterogeneity observed in the results of the review? |
| 15. If they performed quantitative synthesis, did the review authors carry out an adequate investigation of publication bias (small study bias) and discuss its likely impact on the results of the review?***** |
| 16. Did the review authors report any potential sources of conflict of interest, including any funding they received for conducting the review? |
| *****Critical domain |

| Number of columns (number of reviews) | c | 16 |
| --- | --- | --- |
| Number of rows (number of index publications) | r | 104 |
| Number of included primary studies (including double counting) | N | 249 |
| Covered area | N/(rc) | 14.96% |
| Corrected covered area | (N-r)/(rc-r) | 9.29% |
| Interpretation of overlap | Moderate overlap | |
| Structural Zeros | X | 0 |
| Corrected covered area  (adjusting by structural zeros) | (N-r)/(rc-r-X) | 9.29% |

**Supplementary Figure 2. Graphical Representation of Overlap for OVErviews (GROOVE)**


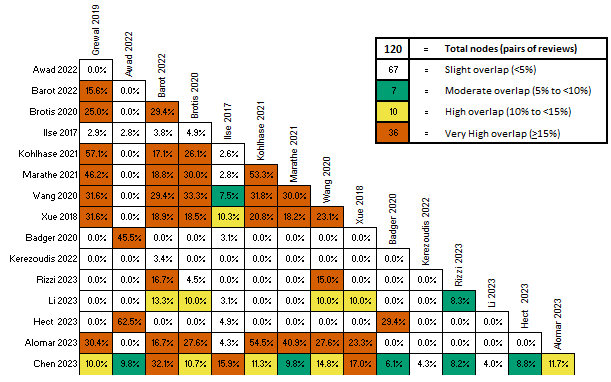


| N° of non-overlapped primary studies | In 1 SR | 47 |
| --- | --- | --- |
| Number of overlapped primary studies | In 2 SRs | 20 |
|  | In 3 SRs | 18 |
|  | In 4 SRs | 6 |
|  | In 5 SRs | 4 |
|  | In 6 SRs | 4 |
|  | In 7 SRs | 1 |
|  | In 8 SRs | 3 |
|  | In 9 SRs | 1 |
|  | In 10 SRs | 0 |
|  | In 11 SRs | 0 |
|  | In 12 SRs | 0 |
|  | In 13 SRs | 0 |
|  | In 14 SRs | 0 |
|  | In 15 or more SRs | 0 |
